# Numerical Optimization of Stress Concentration in Composite Structures for Different Material Arrangement

**DOI:** 10.3390/ma14112957

**Published:** 2021-05-30

**Authors:** Sushant Bhalchandra Pate, Ryszard Korycki

**Affiliations:** 1 Kaunas University of Technology, Donelaičio St., 73, 44249 Kaunas, Lithuania; patesushant95@gmail.com; 2 Department of Technical Mechanics, Informatics and Chemistry of Polymer Materials, Faculty of Material Technologies and Textile Design, Lodz University of Technology, Zeromskiego, 116, 90-924 Lodz, Poland

**Keywords:** numerical optimization, stress concentration, composite structures

## Abstract

Complex machine parts are characterized by different shapes, material characteristics and working loads. The simultaneous theoretical optimization of shape and material properties is difficult because the single objective functional with a unique physical interpretation is unknown for these two features. The optimization is the multi-criteria procedure or the functional is a weighted average of partial criteria with the assumed weight values. Therefore, the structure and material characteristics are optimized numerically. The main goal of the article was to model and optimize the stress distribution inside the composite plates subjected to complex load. The advanced material composition was made of four different materials: steel, ductile iron, E-glass fibers and carbon fibers. The stress distributions were optimized for the homogeneous plate, the sandwich composite made of metal and textile layers, and the plate with additional stiffening elements (ribs and another plate along the neck portion). Based on the numerical simulations, the optimal structural shapes and material arrangements were determined.

## 1. Introduction

Complex machine parts are characterized by different shapes, material characteristics and working loads. The problem can be simplified in the case of the plate of the one dimension (the thickness) being much smaller than the other two. The simultaneous theoretical optimization of shape and material properties is difficult because the single objective functional with a unique physical interpretation is unknown for these two features. This excludes classic optimization using the single objective functional and variational approach. Optimization is a multi-criteria procedure and the functional is a weighted average of partial criteria with the assumed weight values. Thus, the structure and material characteristics should be optimized numerically. The state variable is the stress inside the structure.

The classic optimization problem is solved by means of the Lagrange functional and its stationarity conditions. The mathematical description requires the first-order sensitivities of the objective functional [1]. The variational approach can be applied to optimize/identify the shape and material properties of the elastic structure [2,3]. The structural parameters can be optimized during the coupled heat and moisture transport, for example, to determine the optimal material thickness [4], the thickness in the ironing machine [5,6] and dimensions of seams securing the optimal insulating properties [7].

Basic problems regarding the classic approach, first order problems and lamination plate theory were discussed in [8]. The book [9] presents the theory of plates and shells, which is essential for a structural engineer. Though a number of finite element packages are used by design engineers, one must be proficient in the classic method. The book [10] provides the reader with a consistent approach to theory of structures on the basis of applied mechanics. It covers framed structures, plates and shells, using elastic and plastic theory and the relationship to practical engineering activities. The use of composites in engineering structures continues to increase, and there have been equally significant advances in modeling for general and composite materials and structures in particular [11]. The fundamentals of the finite element method are given in [12], using computer code listings in MATLAB and MAPLE as well as COMSOL files. Emphasis is placed on the development of the discrete set of algebraic equations.

The main goal of the article is to model and optimize the stress distribution in composite plates made of different materials in regard to shape and material properties. The reference structure is a homogeneous metal plate exposed to a constant load. The optimization problem was solved numerically, and the results were compared using different programs (for example Ansys, Catia, etc.), which additionally verifies the correctness of the calculations.

The first phase of calculations allows to compare the stress distributions obtained for the different material arrangements, loads and thicknesses. Based on the preliminary simulations, the optimal material thickness was determined as input data to the further calculations.

Assuming the appropriate dimensions, we introduce the following structures and material arrangements: (i) the complex composite made of several metal and fibrous layers; (ii) the multilayer structure additionally stiffened by ribs in the neck portion; and (iii) the multilayer composite reinforced by an additional plate made of different materials in the neck part. Moreover, the radius was significantly increased to minimize the maximum stress and optimize its distribution inside the concentration zone. The optimal value and distribution of stresses determine the most favorable changes in the structure.

The analysis allows to indicate the following novelty elements: (i) determination of stress distribution for the reference plate of the specified structure and dimensions; (ii) determination of stress distribution in case of additional structural elements securing the most loaded parts; and (iii) optimization of structural shape and material parameters by means of the optimal stress distribution in composite structures with fibrous layer. 

## 2. Problem Definition 

**Figure 1:**
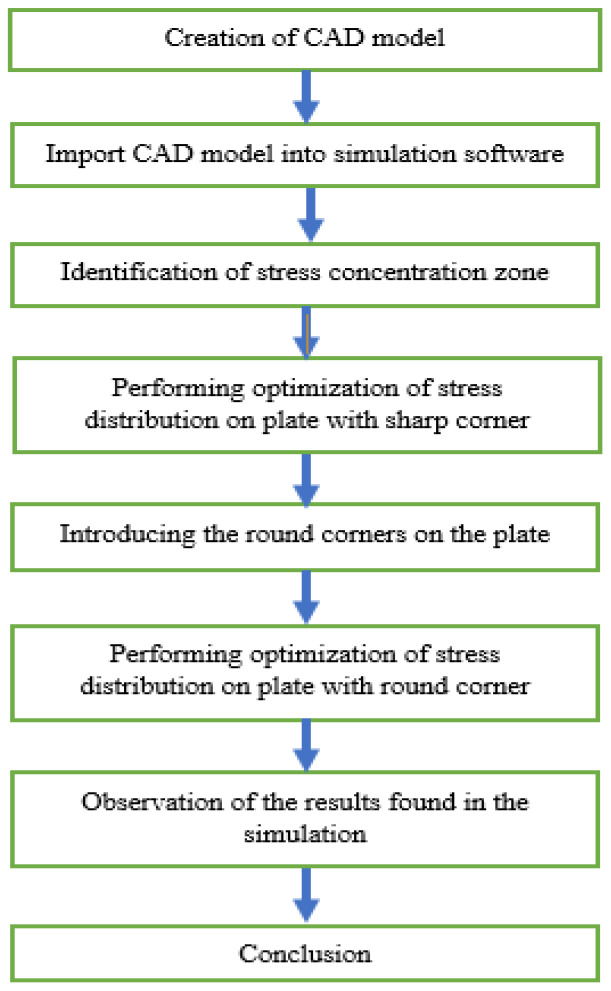
Block diagram of the numerical simulations.

The analyzed structure is a plate of the specified geometry and material characteristics. The stress concentration zone is always located in the upper corner between the neck and the body. The maximum values and distribution of stresses can be optimized by increasing the radius of the sharp edge and introducing additional structural elements (i.e., the sandwich structure made of metal and fiber materials with additional stiffeners). The optimization procedure is shown in Figure 1.

**Figure 2:**
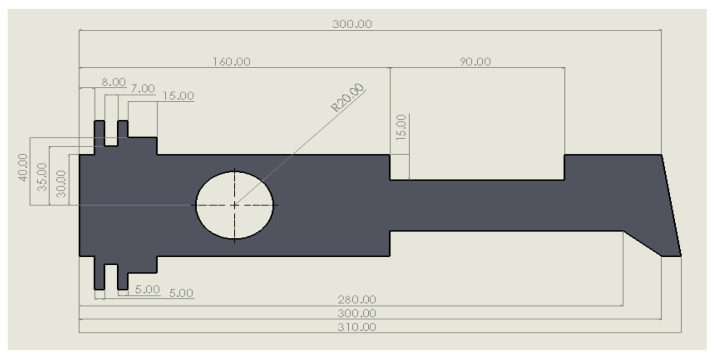
Plate geometry (all dimensions in [mm]).

The plate geometry is presented in Figure 2. Based on the preliminary tests, the material thickness was assumed to be equal to 5 mm.

The structure was made of different materials: two metals and two fibrous materials of the characteristics determined in Table 1. 

**Figure 3:**
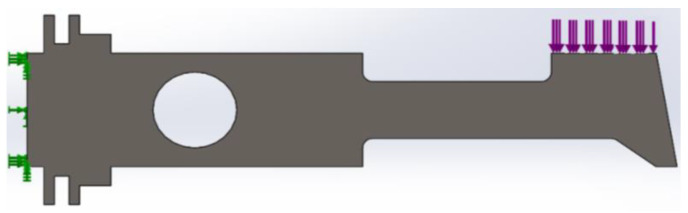
Boundary conditions of plate during numerical simulations.

The plate was subjected to a uniform load at the end equal to 1000 N/m, whereas the other end was fixed (Figure 3). The temperature was assumed to be equal to 300 K.

## 3. Numerical Simulations

**Figure 4:**
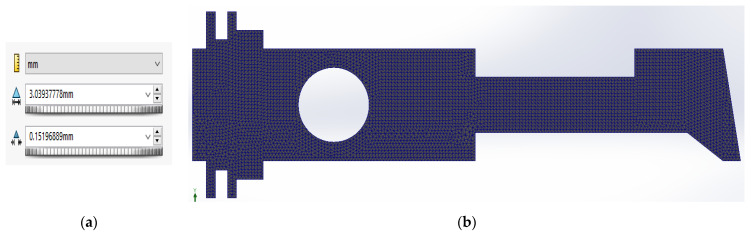
Finite element net; (**a**) mesh size representation; (**b**) example of the finite element net of the plate.

The finite element net applied during the calculations had different sizes of a single element. The structure of the uniform shape, subjected to the insignificant stress gradient was approximated by means of a mesh of average density. In other cases (for example, the circular hole, sharp corners and significant stress gradient) a mesh of minimized density was applied. The advanced mesh allowed to reproduce correctly the structural shape, particularly the diversified geometry or the areas subjected to the substantial gradient of load. The fine and the most efficient mesh net was created using a standard mesh setting with a size of 3 mm with a tolerance of 0.15 mm (Figure 4).

First, the stress distribution was determined to identify the concentration zones on the entire surface (2D problems) and inside the volume (3D problems) of the reference plate. The analysis was limited consequently to areas defined by the maximum stresses. All other places were subjected to lower loads and the material effort was significantly reduced.

**Figure 5:**
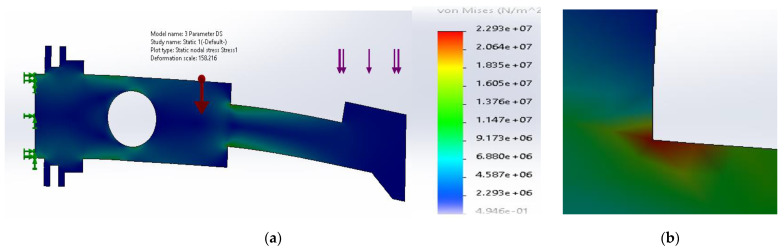
(**a**) Stress distribution according to 2D simulation results for the reference plate; (**b**) zone of maximum stress concentration.

The calculations were carried out using the finite element method. This determines the typical methodology for a complex mesh of FEM. The same distribution of state variable (the stress) in the contact nodes was introduced. Inside the single finite element, the current values were approximated at specific points by means of the nodal shape functions and appropriate weights. The partial values obtained for the single element were next added and the global matrices of the basic FEM equation were formulated. The solution of the global FEM correlation created the distribution of stresses (Figure 5).

The analysis of stress distribution allowed to determine that the stress concentration zone was located in the upper sharp corner between the neck and the body. The maximum stress was significant (2.29 × 10^8^ N/m^2^). Additionally, the stress concentration zone was extensive and covered partially the neck and part of the body. Owing to the establishment of stress distributions it was possible to create a set of 3D models using CAD software (version 2020, Autodesk Inc., San Rafael, CA, USA). The models were introduced into the simulation software to determine the stress fields. 

### 3.1. Metal Plate with Sharp Corners

#### 3.1.1. Uniform Plate Made of SS316

**Figure 6:**
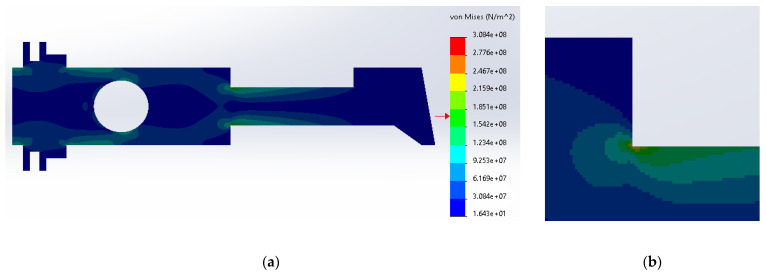
(**a**) Stress distribution according to 3D simulation results for the uniform metal plate made of SS316; (**b**) zone of maximum stress concentration.

Figure 6 shows the distribution and the zone of maximum stress concentration. 

The maximum stresses are concentrated in the upper sharp corner between the neck and the body and are in the range from 3.08 × 10^8^ N/m^2^ to 1.85 × 10^8^ N/m^2^. In the case of the 3D model, the values are considerably greater than inside the plane 2D structure. The stresses are above the yield strength for SS316, which is unacceptable in mechanical structures.

#### 3.1.2. Sandwich Composite Plate Made of Three Layers

**Figure 7:**
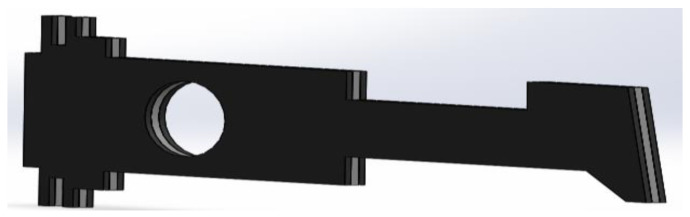
Sandwich composite plate.

**Figure 8:**
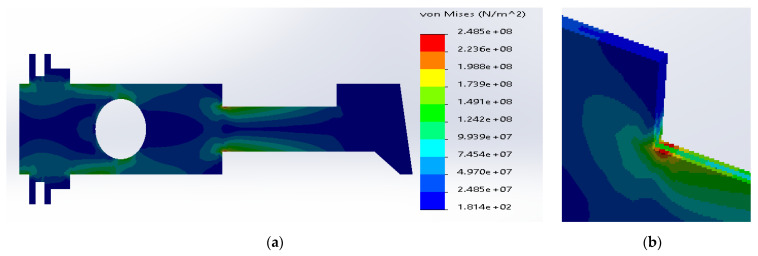
(**a**) Stress distribution according to 3D simulation results for composite plate made of SS316 and ductile iron; (**b**) zone of maximum stress concentration.

**Figure 9:**
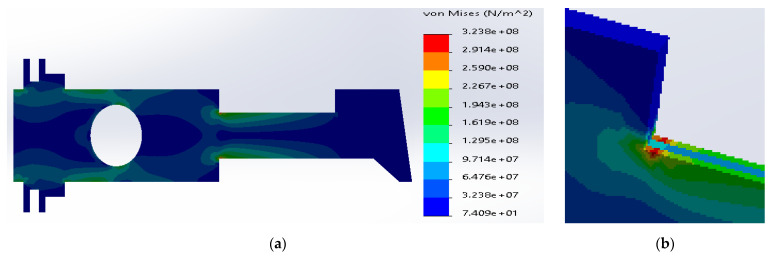
(**a**) Stress distribution according to 3D simulation results for composite plate made of SS316 and E-glass fibers; (**b**) zone of maximum stress concentration.

**Figure 10:**
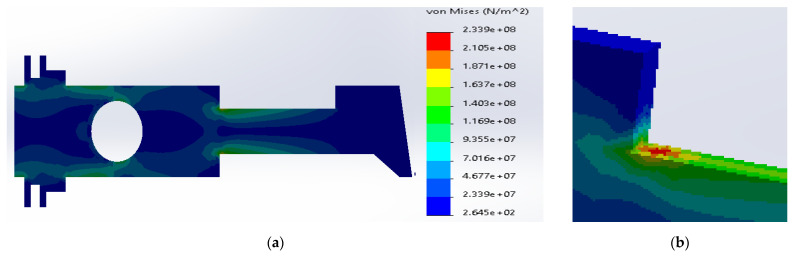
(**a**) Stress distribution according to 3D simulation results for composite plate made of SS316 and carbon fibers; (**b**) zone of maximum stress concentration.

The internal layer made of the different material (i.e., ductile iron, E-glass fibers and carbon fibers) was introduced between two external metal layers made of SS316. The thickness of the single steel element was assumed to be equal to 1.5 mm, whereas the middle part, 2 mm (Figure 7). The stress distributions are shown in Figure 8, Figure 9 and Figure 10.

Based on the simulation results for the composite made of SS316 and ductile iron, the maximum stresses were located inside the metal layers (Figure 8). The value was equal to 3.24 × 10^8^ N/m^2^, which was above the yield stress for SS316. The maximum stress inside the ductile iron (1.24 × 10^+^ N/m^2^) was below the tensile strength for the material.

Similarly, in the case of SS316 and E-glass fibers, the maximum stresses were located inside the metal layers (Figure 9). The current value was considerably reduced to 2.49 × 10^8^ N/m^2^, which is still above the yield strength for SS316. The yield strength of the E-glass fibers was sufficient enough to transfer the stresses.

The most favorable stress distribution was determined for the carbon fibers inside the middle layer, Figure 10. The maximum stresses were defined inside the carbon fibers (2.11 × 10^8^ N/m^2^), which is significantly below the yield strength. The maximum stresses in the metal layer (1.64 × 10^8^ N/m^2^) were below the yield strength for SS316. The above structure can work under the assumed load; the material effort is acceptable.

#### 3.1.3. Uniform Metal Plate with Ribs Introduced in Neck Portion

**Figure 11:**
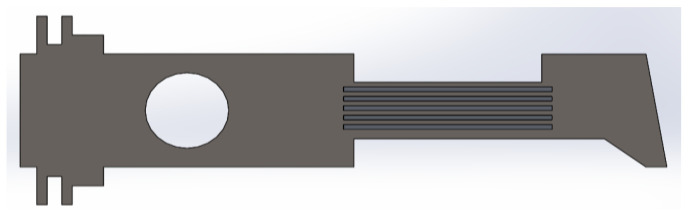
Uniform metal plate with the ribs in neck portion.

Based on the above observations, the structure was modified by five additional ribs of dimensions 100 × 2.5 mm in the neck part, located at a distance of 2.5 mm (Figure 11). The uniform plate is made of steel SS316. The material of the ribs is different.

**Figure 12:**
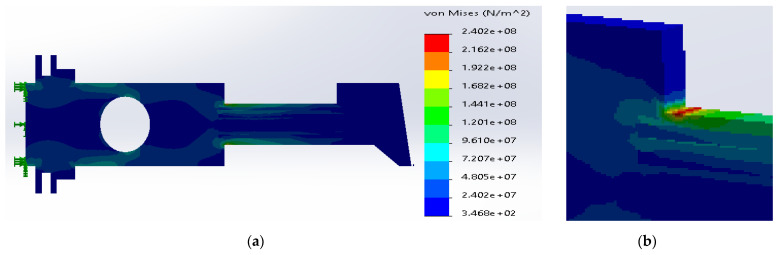
(**a**) Stress distribution according to 3D simulation results for SS316 metal plate with the ductile iron ribs; (**b**) zone of maximum stress concentration.

**Figure 13:**
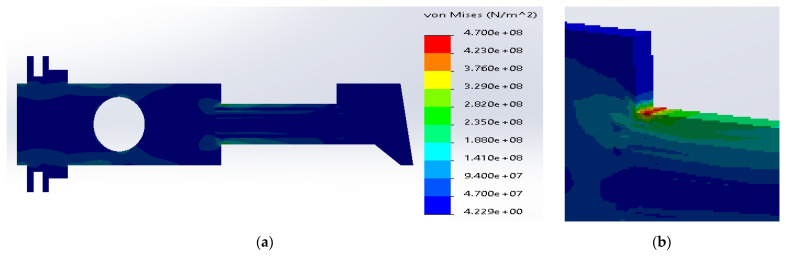
(**a**) Stress distribution according to 3D simulation results for SS316 metal plate with the E-glass fiber ribs; (**b**) zone of maximum stress concentration.

**Figure 14:**
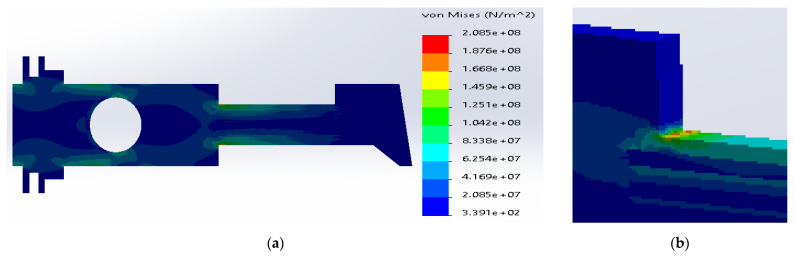
(**a**) Stress distribution according to 3D simulation results for SS316 metal plate with the carbon fiber ribs; (**b**) zone of maximum stress concentration.

The simulation results for different materials are shown in Figure 12, Figure 13 and Figure 14. 

According to Figure 12 and Figure 13, the obtained distributions were similar for the ribs made of ductile iron and E-glass fibers. The carbon fibers generated the different character of stresses along the thickness of plate, Figure 14. However, the maximum stresses are still above the yield strength, which is destructive for the material.

#### 3.1.4. Uniform Metal Plate with Additional Rectangular Plate Introduced in Neck Portion

**Figure 15:**
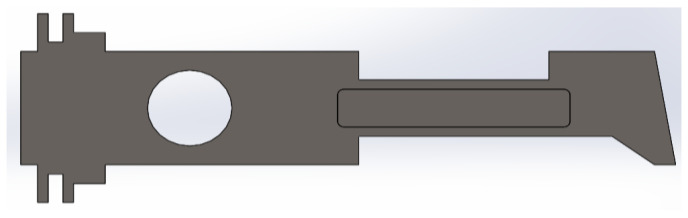
Metal plate with rectangular plate introduced in neck portion.

**Figure 16:**
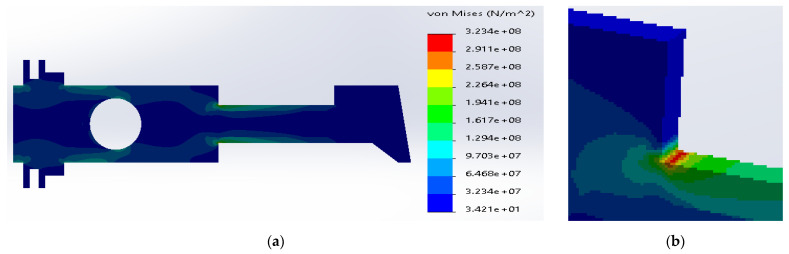
(**a**) Stress distribution according to 3D simulation results for SS316 metal plate with additional plate made of ductile iron; (**b**) zone of maximum stress concentration.

**Figure 17:**
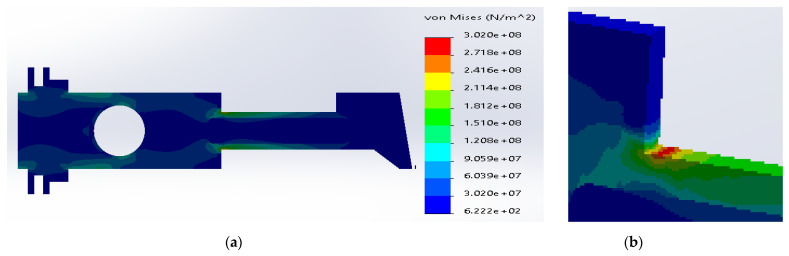
(**a**) Stress distribution according to 3D simulation results for SS316 metal plate with additional plate made of E-glass fibers; (**b**) zone of maximum stress concentration.

**Figure 18:**
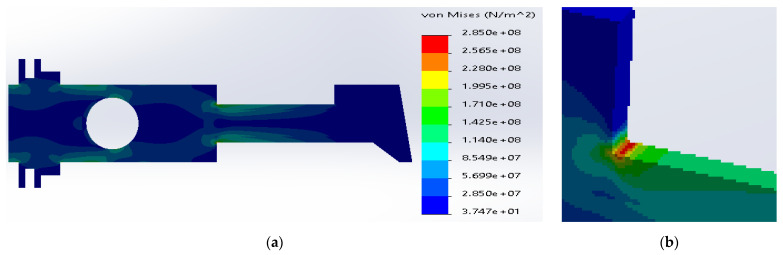
(**a**) Stress distribution according to 3D simulation results for SS316 metal plate with additional plate made of carbon fibers; (**b**) zone of maximum stress concentration.

In this case, the additional rectangular plate of dimensions 100 × 20 mm, located in the neck of the uniform metal plate, was made of SS316 (Figure 15). The simulation results for the additional plate made of different materials are shown in Figure 16, Figure 17 and Figure 18. 

The analysis of stress distributions allowed to determine that the introduction of an additional plate does not cause any significant changes in the concentration zone. The only change is a slight shift of the maximum stresses toward the neck of the structure. However, the maximum stresses were comparable to the previous maximum values; all were above the yield strength. 

### 3.2. Uniform Metal Plate with Rounded Corners

**Figure 19:**
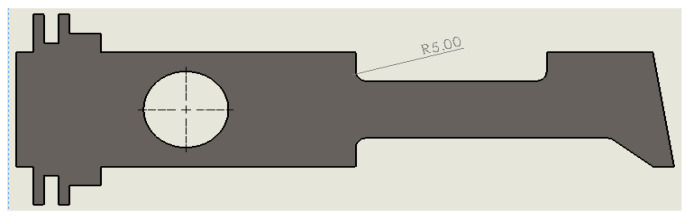
Modified metal plate with rounded corners R 5 mm.

The source of significant stress were the sharp corners at the contact surface between the body and the neck. Instead of a negligible radius of sharp edge R→0, its value increased to R 5 mm, Figure 19. The particular value of the radius allowed to evaluate the plate in regard to the entire stress distribution in the concentration zone.

#### 3.2.1. Uniform Plate Made of SS316

**Figure 20:**
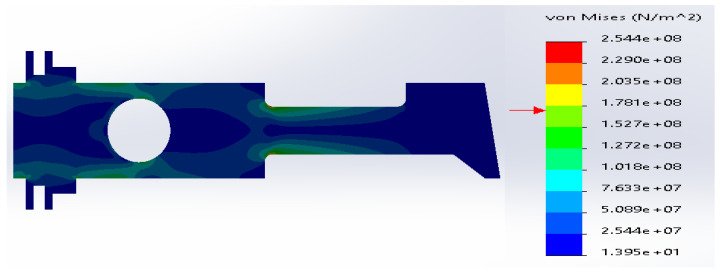
Stress distribution according to 3D simulation results for the uniform metal plate made of SS316 and R 5 mm.

The maximum stresses in the upper corner between the neck and the body were concentrated along the neck of the plate as well as along the rounding edge (Figure 20). The maximum values varied along the edge between 2.5 × 10^8^ N/m^2^ and 1.6 × 10^8^ N/m^2^. Although the stresses were less than in the previous case R→0, the values were greater than the yield strength.

#### 3.2.2. Sandwich Composite Plate Made of Three Layers

**Figure 21:**
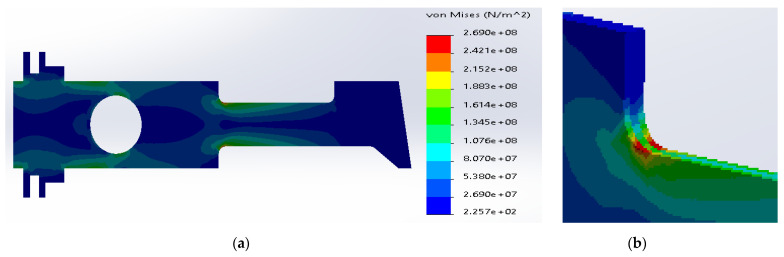
(**a**) Stress distribution according to 3D simulation results for composite plate made of SS316 and ductile iron, R 5 mm; (**b**) zone of maximum stress concentration.

**Figure 22:**
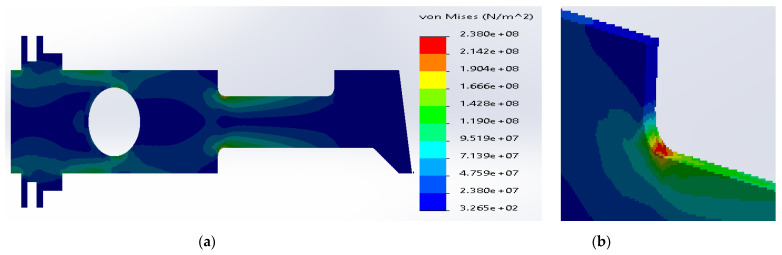
(**a**) Stress distribution according to 3D simulation results for composite plate made of SS316 and E-glass fibers, R 5 mm; (**b**) zone of maximum stress concentration.

**Figure 23:**
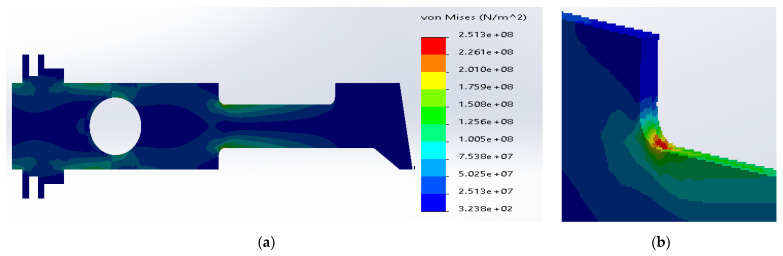
(**a**) Stress distribution according to 3D simulation results for composite plate made of SS316 and carbon fibers, R 5 mm; (**b**) zone of maximum stress concentration.

The sandwich plate is defined in Section 3.1.2. The obtained stress distributions are shown in Figure 21, Figure 22 and Figure 23.

Introducing the sandwich composite structure, the reduced value of maximum stresses was obtained. In the case of the middle layer made of ductile iron (Figure 21), the maximum stresses in SS316 were higher than the yield strength (2.41 × 10^8^ N/m^2^), whereas inside the ductile iron, were equal to 3.05 × 10^8^ N/m^2^. Thus, the composite structure remained continuous and integral under load.

The structure made of SS316 and E-glass fibers induced reduced stress in the metal layer equal to 2.1 × 10^8^ N/m^2^ (Figure 22). Moreover, the maximum stresses were determined in the fibrous layer of the values below the yield strength.

The most advantageous results were obtained for the structure made of SS316 and carbon fibers. The stress zone is reduced significantly and concentrated inside the fibrous layer, which is defined by the much greater yield strength than steel. The stress values in the metal layer are slightly above the yield strength of the material (2.03 × 10^8^ N/m^2^).

Owing to the results of the simulations, it is possible to predict that these solutions are acceptable in regard to the maximum stress distribution inside the materials.

#### 3.2.3. Uniform Metal Plate with Ribs Introduced in Neck Portion

**Figure 24:**
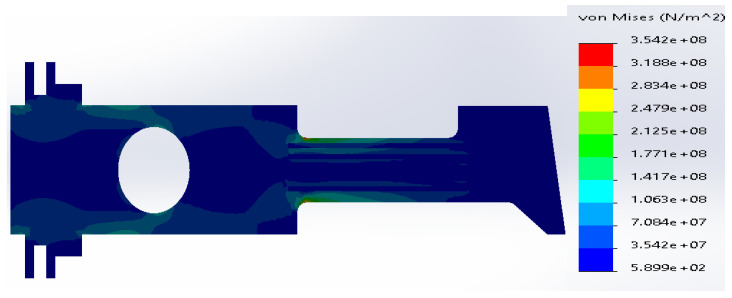
Stress distribution according to 3D simulation results for SS316 metal plate with the E-glass fiber ribs, R 5 mm.

**Figure 25:**
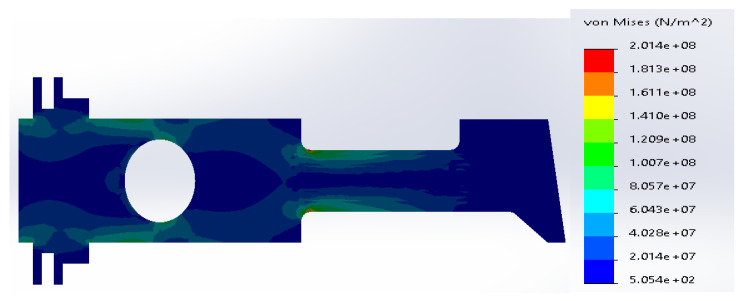
Stress distribution according to 3D simulation results for SS316 metal plate with the carbon fiber ribs, R 5 mm.

The plate is defined in Section 3.1.3. The only difference is the radius of sharp edge R 5 mm. The obtained stress distributions are shown in Figure 24 and Figure 25.

The use of ribs allowed to optimize the distribution of stress concentrations, particularly in the neck portion. According to the above figures, the values of maximum stresses were considerably limited, and the distributions were beneficial. The maximum stresses were concentrated along the neck of the plate. The stress concentrations were comparable for the ribs made of ductile iron and E-glass fibers, (cf. Figure 24).

The best distribution of stresses was observed for the ribs made of carbon fibers. The maximum stress is reduced and close to the yield strength level for steel. This solution can be assessed as acceptable in composites, despite the considerable material effort.

#### 3.2.4. Uniform Metal Plate with Additional Rectangular Plate Introduced in Neck Portion

**Figure 26:**
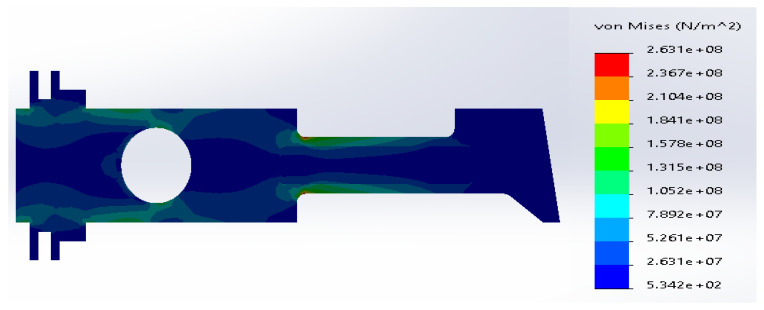
Stress distribution according to 3D simulation results for SS316 metal plate with additional plate made of ductile iron, R 5 mm.

**Figure 27:**
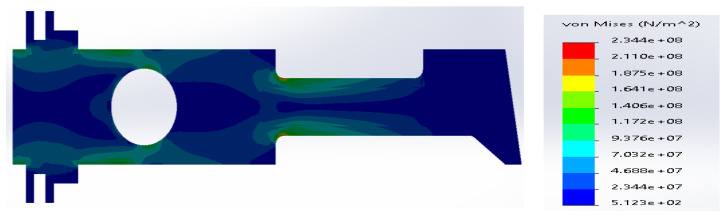
Stress distribution according to 3D simulation results for SS316 metal plate with additional plate made of carbon fibers R 5 mm.

The material and geometric parameters are defined in Section 3.1.4, but the radius of the sharp edge is equal to R 5 mm. The stress distributions obtained during simulations are shown in Figure 26 and Figure 27.

Regardless of the material of the additional plate, the stress distributions obtained during the calculations are comparable. In case of ductile iron and E-glass fibers, the changes are negligible—the maximum values and their distributions are almost the same. Therefore, only Figure 26 for the additional plate made of ductile iron is demonstrated.

In the case of the additional plate made of carbon fibers, the maximum value is practically the same as for sharp corners R→0 but the stress was distributed along the neck.

In summary, the uniform metal plate of the rounded corners R 5 mm with an additional rectangular plate ensures the acceptable stress distribution for the analyzed composite structure.

## 4. Conclusions

In the case of complex composite structures, application of the classic, variational approach of an optimization procedure can be unrealizable. The simultaneous theoretical optimization of shape and material properties is difficult because the single objective functional of a unique physical interpretation is unknown for these two features. The procedure is the multicriteria optimization, or the objective functional is determined as a weighted average of partial criteria with the assumed weight values.

Therefore, we presented the optimization approach, introducing the numerical analysis of the different structural variants. The reference structure was always the uniform metal plate, whereas the modifications included the following versions: (i) the sandwich composite structure, (ii) the additional ribs along the neck, and (iii) the additional plate covering the neck of the structure. The complex material composition was made of four different materials: steel, ductile iron, E-glass fibers and carbon fibers. Irrespective of the structure, shape and material composition, the maximum stresses were above the acceptable yield strength level. The zone of maximum stress concentration was always located around a sharp corner with an extremely small radius and along the neck. The only exception was the sandwich structure made of steel and carbon fibers inside the middle layer. The maximum stresses in both layers were below the yield strength for the corresponding material.

To reduce the maximum stress and improve its distribution, it was necessary to increase the radius of the sharp corners to the value of R 5 mm. The particular value of the radius allowed to evaluate the plate in regard to the entire stress distribution in the concentration zone. Thus, the calculation procedure was next repeated. The best stress distribution was obtained for the sandwich structure made of three layers: the external steel SS316 and the internal carbon fibers (the most effective material in regard to tension). The maximum stress was minimized, and the stress concentration zone was reduced and distributed advantageously inside the material.

The disadvantage of this approach is a large number of consecutive numerical approximations. The method is consequently time and labor consuming. Nevertheless, it is the only known procedure that allows to optimize both shape and material properties and determine the optimal stress inside a non-homogeneous plate subjected to complex loads.

The maximum stress concentration was analyzed for the plate with one additional element (i.e., the sandwich composite structure, the additional ribs along the neck, the additional plate in neck portion). The advanced optimization method requires the application of more than one structural solution. This approach is, consequently, much more complicated and labor consuming. On the other hand, the calculations are more efficient, relatively fast and can determine the global minimum of stresses in the concentration zone.

## Figures and Tables

**Table 1:** Characteristics of materials applied.

Material	Mass Density kg/m^3^	Elastic Modulus 10^10^ N/m^2^	Yield Strength 10^8^ N/m^2^	Tensile Strength 10^8^ N/m^2^	Poisson’s Ratio
SS316	8000	19.29	1.72	5.80	0.27
Ductile iron	7200	6.62	-	1.52	0.27
Carbon fiber	2580	7.23	178	34.45	0.2
E-glass fiber	1760	23.00	3.61	35.30	0.23

## Data Availability

Not applicable.
